# From Genes to -Omics: The Evolving Molecular Landscape of Malignant Peripheral Nerve Sheath Tumor

**DOI:** 10.3390/genes11060691

**Published:** 2020-06-24

**Authors:** Kathryn M. Lemberg, Jiawan Wang, Christine A. Pratilas

**Affiliations:** 1 Sidney Kimmel Comprehensive Cancer Center at Johns Hopkins, Baltimore, 401 N Broadway, Baltimore, MD 21231, USA; klember1@jhmi.edu (K.M.L.); jwang255@jhmi.edu (J.W.); 2 Johns Hopkins University School of Medicine, Baltimore, 733 N Broadway, Baltimore, MD 21205, USA

**Keywords:** MPNST, NF1, genomics

## Abstract

Malignant peripheral nerve sheath tumors (MPNST) are rare, aggressive soft tissue sarcomas that occur with significantly increased incidence in people with the neuro-genetic syndrome neurofibromatosis type I (NF1). These complex karyotype sarcomas are often difficult to resect completely due to the involvement of neurovascular bundles, and are relatively chemotherapy- and radiation-insensitive. The lifetime risk of developing MPNST in the NF1 population has led to great efforts to characterize the genetic changes that drive the development of these tumors and identify mutations that may be used for diagnostic or therapeutic purposes. Advancements in genetic sequencing and genomic technologies have greatly enhanced researchers’ abilities to broadly and deeply investigate aberrations in human MPNST genomes. Here, we review genetic sequencing efforts in human MPNST samples over the past three decades. Particularly for NF1-associated MPNST, these overall sequencing efforts have converged on a set of four common genetic changes that occur in most MPNST, including mutations in neurofibromin 1 (*NF1*), *CDKN2A*, *TP53*, and members of the polycomb repressor complex 2 (PRC2). However, broader genomic studies have also identified recurrent but less prevalent genetic variants in human MPNST that also contribute to the molecular landscape of MPNST and may inform further research. Future studies to further define the molecular landscape of human MPNST should focus on collaborative efforts across multiple institutions in order to maximize information gathered from large numbers of well-annotated MPNST patient samples, both in the NF1 and the sporadic MPNST populations.

## 1. Clinical Overview of MPNST

MPNST are aggressive soft tissue sarcomas originating from Schwann cells in the peripheral nervous system [1,2]. Half of MPNST occur in patients with the cancer predisposition syndrome NF1, caused by germline loss of function (LOF) of one copy of the tumor suppressor gene *NF1*. In patients with NF1, most MPNST arise from within plexiform neurofibromas (pNF), which are pre-malignant tumors of the peripheral nerve [3,4,5]. pNF can, themselves, be a major source of disfigurement or dysfunction. MPNST can also occur sporadically or following radiation treatment in the general population, although the incidence of the latter is substantially lower. MPNST carry a high risk of sarcoma-specific death; in the absence of complete surgical resection with wide negative margins, the five-year event-free survival is ~30% [6,7]. Conventional chemotherapy and radiation often do not improve patient outcomes [8].

## 2. Germline Loss of NF1: Correlations with NF1 Phenotype 

NF1 is one of the most common monogenic inherited syndromes with an incidence of approximately 1:3000 live births [9]. This neurocutaneous syndrome is characterized by several hallmark skin findings (café au lait macules, axillary freckling, cutaneous neurofibromas), may involve additional organ systems (including CNS, musculoskeletal, and vascular manifestations) [10], and predisposes patients to an increased risk of malignancy, with an estimated lifetime cancer risk ~60% [11]. One of the hallmark lesions in NF1 patients is the pNF, a complex lesion that grows along major nerve bundles. While benign, pNF can result in significant anatomic, functional, cosmetic, and psychological effects [12]. In patients with NF1, MPNST may arise within existing pNF and are often accompanied by rapid growth, increased pain, or other nervous system deficits. Studies correlating the pathologic changes and genetic alterations in the peripheral nerves of NF1 patients or model organisms, along the spectrum from healthy to pNF to atypical neurofibroma (ANF) to MPNST, have aided in understanding the roles of specific genetic mutations in MPNST tumorigenesis [13]. 

**Figure 1:**
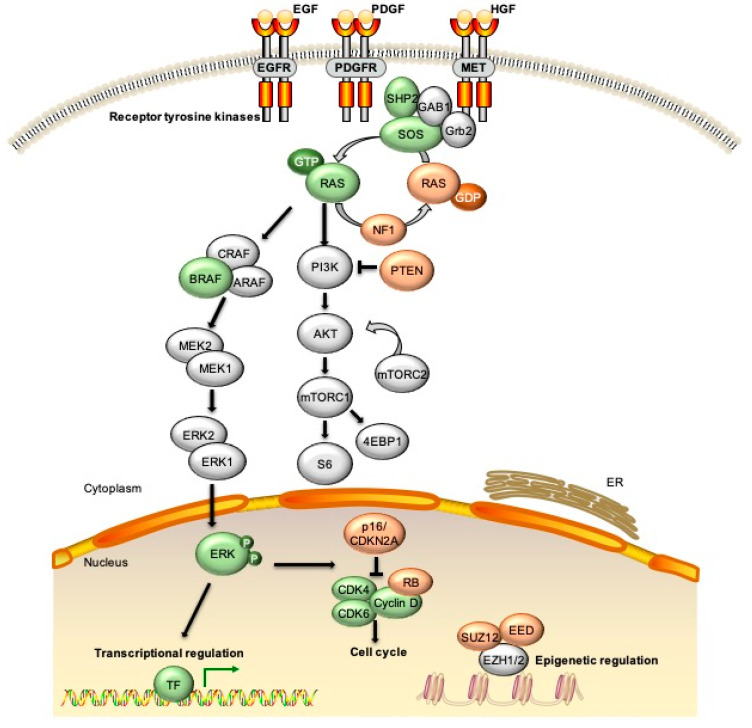
Signaling pathways altered due to genetic changes observed in malignant peripheral nerve sheath tumors (MPNST). The most common alterations in MPNST are loss of function of multiple tumor suppressors including NF1, p16/CDKN2A, TP53, and SUZ12/EED. Loss of *NF1*, as well as epigenetic changes due to loss of PRC2 components, leads to increased signaling through the RAS/RAF/MEK and PI3K/AKT pathways. Additional molecular events observed in subsets of MPNST include mutations in *BRAF*, amplification of *EGFR* or *MET* receptor tyrosine kinases (RTKs), and changes to chromatin structure through mutations in alpha thalassemia/mental retardation syndrome X (ATRX) and other epigenetic modifiers. EGF/EGFR = epidermal growth factor/receptor; PDGF/PDGFR = platelet derived growth factor/receptor; HGF = hepatocyte growth factor; ERK = extracellular signal regulated kinase; CDK = cyclin dependent kinase; RB = retinoblastoma; TF = transcription factor; ER = endoplasmic reticulum.

NF1 syndrome is characterized by a wide variation in phenotypic expression which partially reflects the large number of mutations in the *NF1* gene that have been identified in people with the condition [14,15,16]. The *NF1* gene was originally cloned nearly three decades ago [17]. It is a large gene, approximately 350 kb in length, located on human chromosome 17q11.2. There may be multiple splice variants [18] but the primary gene product codes for the NF1 protein of 2,818 amino acids, which acts as a GTPase-activating protein (GAP) for RAS oncogenes [19,20,21,22]. Loss of *NF1*, therefore, leads to constitutive activation of RAS signaling [23,24] (Figure 1), likely accounting for the pro-tumor phenotype observed in patients with NF1 [25].

Historically, the diagnosis of neurofibromatosis was based on clinical symptoms and physical findings, without a requirement for clinical genetic testing [26]. With the advances in detailed sequencing efforts, however, disruption of a copy of *NF1* in the germline may be identified in the majority of patients with NF1 [27]. Mutational analysis demonstrates a very high rate of mutations occurring in *NF1*, as evidenced by the fact that approximately 50% of cases of NF1 appear to be *de novo*. To date, hundreds of mutations associated with the syndrome have been characterized [9,14]. Identification of the specific LOF mutation in patients can be helpful for testing family members, particularly offspring of those affected, and for counseling patients about syndrome-specific risks. 

Genotype–phenotype correlations associated with specific germline *NF1* alterations have been observed in a limited number of cases. Two examples are associated with limited risk for MPNST. A small in-frame deletion (c.2970_2972del(p.Met992del)) leading to loss of a methionine in the cysteine-serine rich domain (CSRD) of *NF1* is associated with suppression of cutaneous neurofibroma (cNF) and clinically apparent pNF formation, though these individuals have an increased risk for learning disabilities (48%) and brain tumors (~5%) [28,29]. Several missense mutations affecting arginine 1809 (e.g. p.Arg1809Cys) have also been characterized in multiple unrelated families. These patients have a high prevalence of developmental delay and learning disabilities as well as short stature and pulmonic stenosis, but few cutaneous or plexiform neurofibromas, and low risk of malignancy [30].

By contrast, two other *NF1* genotypes have been strongly associated with a higher risk of MPNST. Microdeletion of a 1.4 Mbp segment of chromosome 17 due to homologous recombination within duplication regions of the chromosome leads to deletion of 14 functional genes [31,32]. Individuals with the microdeletion syndrome (approximately 5% of NF1 cases) tend to present with a more severe NF1 phenotype [33], including dysmorphic features, developmental delay, intellectual disability, increased number of neurofibromas, and a two-fold higher lifetime risk for MPNST (16–26%, compared to approximately 8–13% risk in the general NF1 population) [34,35]. Missense mutations in NF1 protein codons 844–848 (including Leu844, Cys845, Ala846, Leu847, and Gly848; located in the CSRD) occur in ~0.8% of studied NF1 cases and are also reported as a risk factor for severe phenotypic presentation. These patients have higher numbers of clinically apparent major pNF, symptomatic spinal neurofibromas, optic pathway gliomas, and skeletal abnormalities, and up to 10% develop malignancy, including MPNST [36]

## 3. Sequencing Efforts in Human MPNST Samples: Improvements in Technology with Variability in Study Design

A collated summary of human MPNST sequencing efforts over the past two decades is shown in Table 1. MPNST have complex karyotypes with multiple chromosomal losses and gains and structural anomalies; a single recurrent translocation for diagnostic purposes has not been defined for MPNST as it has for some other mesenchymal tumors [37]. Expanded knowledge of MPNST gene alterations originated in the era of targeted gene evaluation using sequencing specific to the *NF1* locus or a small number of related genes. More recent studies have employed whole exome, whole genome, or targeted next-generation sequencing (NGS) on discovery cohorts for MPNST, with follow up studies performed by targeted gene sequencing in validation cohorts. Whole exome sequencing (WES) efforts have also been performed on patient tumors with paired neurofibroma or blood samples in a minority of cases. Individual studies vary with respect to how much additional clinical information is available (e.g., clinical background, treatment effect, comparison to neurofibroma or blood leukocytes). Some studies include sporadic and radiation-associated cases, while others focus purely on NF1-associated MPNST. In addition, in several studies multiple MPNST samples are derived from the same patient or fragments of the same tumor. These differences in study design, sample collection and annotation, and data analysis likely account for some of the differences and depth of discovery in genomic alterations across the literature. Taken together, however, a clear picture emerges of several characteristic alterations (i.e., *CDKN2A*, genes encoding PRC2 components) involved in evolution of benign nerve sheath tumor to MPNST. Less frequent alterations (i.e., *BRAF*, *MET*) identified in smaller subsets also merit additional attention in follow up evaluations, particularly as new diagnostic and treatment strategies for these tumors are being developed.

## 4. Somatic NF1 Mutations in Tumors Including MPNST

Consistent with its role as a classical tumor suppressor gene, loss of heterozygosity (LOH) or “second-hit” somatic mutations in the inherited wild-type *NF1* allele have been detected in a variety of tumors in patients with NF1, including pheochromocytomas [50], breast cancer [51], and hematologic malignancies [52]. Somatic LOH analysis using PCR markers performed on the *NF1* locus in dermal neurofibromas identified deletions in a subset of tumors in several early studies [53,54]; those cases known to be familial were analyzed further and shown to have deletions in the non-germline allele, demonstrating that somatic inactivation of *NF1* occurs in these benign lesions. Several studies have compared germline and somatic *NF1* mutations in MPNST. In a single study which investigated 34 MPNST from 27 NF1 patients, germline mutations were identified by lymphocyte DNA in 22 cases—these included one large 1.4 Mbp genomic deletion, one two-exon deletion, and smaller mutations (missense, nonsense, frameshift, and splicing anomalies) in the remainder [55]. In the same cohort, somatic *NF1* mutations were identified in 31 out of 34 MPNST samples—of these, 28 (91% tumors) were large genomic deletions that partially or entirely deleted the *NF1* gene. The authors speculate that in some cases somatic *NF1* mutations arise upon aberrant intrachromosomal recombination of the *NF1* gene during mitosis. Similarly, another report screened 47 MPNST from patients with or without NF1 syndrome (*n* = 25 and 22 cases, respectively). Of the somatic *NF1* mutations identified (*n* = 10/25 NF1-associated and 9/22 sporadic), approximately 55–60% involved large genomic copy number changes (i.e., deletions) in both NF1 and sporadic MPNST [32]. By contrast, in MPNST analyzed from NF1 patients with the 1.4 Mbp germline *NF1* microdeletion, the *NF1* somatic hit is typically a small (e.g., missense) mutation [31]. Interestingly, in a single patient with clinical NF1 syndrome who developed asynchronous cNF, a primary breast tumor, and later gluteal MPNST, WES revealed three distinct *NF1* somatic mutations compared to the germline mutation noted in the blood [51]. 

## 5. Acquired Mutations during Transformation from pNF

### 5.1. Loss of CDKN2A/B: Correlations with the pNF to ANF Transition

*NF1* LOH is considered to be an initiating event in pNF formation as confirmed in several animal models [56]. Several additional mutations are necessary for malignant transformation. ANF (now re-classified as atypical neurofibromatous neoplasms of uncertain biological potential, ANNUBP) are precursor lesions to NF1-associated MPNST, representing an intermediate step from the malignant transformation of pNF into MPNST [57,58,59]. Alterations to chromosome 9q have been observed in a high proportion of ANF and MPNST [48,60]; one study noted deletion at 9p21.3, identified in 94% (15/16) of ANF and in 70% (16/23) of high-grade MPNST but not in pNF [57]. This locus encompasses several candidate tumor suppressors, including *CDKN2A/B*. *CDKN2A* encodes two gene products each the result of differential splicing: p16^ink4a^ (a negative regulator of CDK4 and CDK6 cyclin dependent kinases) and p19^Arf^, a negative regulator of the *TP53* E3 ligase MDM2. Several early studies on human NF1-associated MPNST specimens identified deletions within the short arm of chromosome 9, in the region of *CDKN2A*, as well as low expression of p19, while these were not detected in neurofibroma samples [61,62]. A more recent study identified frequent somatic deletions of *CDKN2A/B* (69%) and *SMARCA2* (42%), apart from recurrent *NF1* somatic mutations (81%), in 16 ANF [48]. These studies indicate that *CDKN2A/B* deletion is the first step in the progression of pNF toward ANF and eventually MPNST.

### 5.2. LOH and Mutation in the Tumor Suppressor TP53: Not Universal in Human MPNST

Copy number variation and mutations in the tumor suppressor gene *TP53* have been identified in some cases of NF1-associated MPNST. Early studies on small subsets of NF1-associated neurofibrosarcomas identified deletions on chromosome 17 outside of the *NF1* locus [63,64], which included the coding region for *TP53*. Screening for *TP53* inactivation in a panel of 20 MPNST identified LOH in over half of the tumors tested [55]. The first genetically-engineered mouse (GEM) model for MPNST made use of LOH of both *NF1* and *TP53* from mouse chromosome 11 as the tumor initiating event [65]. Numerous subsequent studies have focused on identifying the true incidence of *TP53* mutation in human MPNST; from compiled data on 25 studies including 114 MPNST (both NF1-associated and sporadic), *TP53* mutations were observed in 14% of MPNST, with LOH in 39% of cases (Table 1) [39]. WES of NF1 tumor samples from a single patient with pNF, MPNST, and metastatic sites also identified loss of one copy of *TP53* in the MPNST and metastatic lesion, but not the primary pNF [66]. Genetic changes in *TP53* are thus present in some MPNST but not necessary for all cases of pNF malignant transformation.

### 5.3. Loss of PRC2 or H3K27me3: Recurrently and Specifically Occurs in MPNST

Components of the epigenetic regulatory PRC2are recurrently and specifically inactivated in MPNST (Table 1). Chi and colleagues identified genomic alterations in *EED* (37%, or 19/52) and *SUZ12* (48% or 25/52) in MPNST, alongside frequent somatic alterations in *CDKN2A* (81%, 42/52) and *NF1* (87%, 45/52) [43]. Bettegowda and colleagues simultaneously reported PRC2 loss via *EED* (2%, 1/50) and *SUZ12* (32%, 16/50) mutations in 50 MPNST [42]. De Raedt et al. similarly reported alterations in *EED* in 29% (15/51) and *SUZ12* in 63% (32/51) of NF1-associated MPNST [41]. PRC2-component loss in MPNST is associated with complete loss of histone H3 trimethylation at lysine 27 (H3K27me3) and increased level of H3K27 acetylation (H3K27Ac), which can serve as biomarkers to improve upon the accuracy of the diagnosis of MPNST [41,43]. *SUZ12* loss potentiates the effects of NF1 loss by amplifying RAS-driven transcription through effects on chromatin that triggers an epigenetic switch [41]. Further detail on the role and function of PRC2 elements in MPNST is found in the review article by Zhang et al. dedicated to this topic, also included in this Special Issue on *Genomics and Models of Nerve Sheath Tumors* [67]. Collectively, the highly recurrent and specific inactivation of PRC2 components, *NF1*, and *CDKN2A/B* posits their critical and potentially cooperative roles in MPNST pathogenesis.

## 6. Less Common Recurrent Variants Identified with Modern Sequencing Investigations of MPNST

MPNST demonstrate complex genomic imbalances and chromosomal aberrations [58,59]. In addition to the common deletions of tumor suppressor genes *NF1*, *CDKN2A*, *TP53* and LOF in the PRC2 genes *EED* and *SUZ12*, several other recurrent genomic events have been identified in NF1-associated and sporadic MPNST. Significant findings from these studies are highlighted in Table 2 and described below.

### 6.1. BRAF Mutation: An Alternate Mechanism for Activation of RAS Signaling

In addition to loss of *NF1* and PRC2function, *BRAF* mutations are reported as an alternate mechanism for aberrant activation of RAS signaling in MPNST, albeit at a lower frequency (ranging from 0–9.7%) [32,47,69,78,79], and occurring more commonly in sporadic than NF1-associated cases [78]. Strongly activating kinase mutations (BRAF V600E) occurred in five out of ten *BRAF*-mutant *NF1*-wild type MPNST (*n* = 84; Table 2) [47]. *BRAF* amplification has also been described, with a frequency of 31% in another study cohort consisting of 51 MPNST [40]. Brohl et al. suggest that the relative strength of RAS-activating mutations may determine whether *BRAF* and *NF1* mutations (or *NRAS*/*KRAS* and *NF1*) co-occur and thereby serve together to result in ERK signaling hyperactivation [45]. 

### 6.2. EGFR, MET and Other Receptor Tyrosine Kinases: Frequent Copy Number Gains in MPNST

A variety of oncogenic receptor tyrosine kinases (RTK) are frequently altered in MPNST. In MPNST, alterations in RTK usually take the form of amplification, rather than single nucleotide variations that result in constitutively activated kinases (Table 2). Several early aCGH studies revealed amplifications of *HGF*, *MET*, *EGFR*, *PDGFRA*, and *IGF1R* in approximately 25% to 40% of analyzed MPNST [38,40]. These studies and others [71,72,80] suggest a putative role of these genes and their respective biological pathways in the initiation and/or progression of MPNST. 

Notably, *HGF* and its receptor *MET*, co-located at chromosome 7q, are highly expressed in a relatively large panel of human MPNST samples, and increased phospho-MET expression level directly correlates with shorter MPNST patient survival [81]. A single patient study revealed progressive amplifications of *HGF*, *MET* and *EGFR* in a patient with MPNST harboring early *NF1* and *TP53* loss, using longitudinal genomic analysis from pNF, to MPNST, to metastatic recurrence. These studies further justify investigation of the role of RTK signaling, in particular HGF/MET, on the progression of MPNST.

### 6.3. AURKA Amplification

Dramatic upregulation (7.9-fold) of *AURKA* (the gene encoding aurora kinase A) was observed through RAS-driven transcriptome analysis on a GEM model and 14 human MPNST samples compared with normal nerves. Further analysis using SNP-array and qPCR confirmed copy number gains in the *AURKA* locus in eight out of 13 primary MPNST and five out of five MPNST cell lines but not neurofibromas [74]. Reducing the expression and activity of Aurora kinase using shRNA knockdown and a kinase inhibitor MLN8237, respectively, inhibits MPNST cell survival in vitro and in vivo, and supports the role of aurora kinase as a rational therapeutic target for MPNST [82].

### 6.4. Tyrosine Kinase 2 Overexpression in MPNST 

NGS on a set of seven NF1-associated MPNST identified a predicted pathogenic mutation in tyrosine kinase 2 (*TYK2*) in two out of seven tumors [75]. TYK2 P1104A mutated tumors demonstrated strong immunoreactivity, whereas TYK2 wild type tumors were not immunoreactive. Strong TYK2 expression as assayed by immunohistochemical staining was observed in 63% of MPNST in an independent tissue set, while only 11% of pNF samples stained for TYK2. Ablation of TYK2 expression in human and murine MPNST cells resulted in increased cell death in vitro and decreased tumor growth in a murine model [83]. The example of *TYK2* suggests the role that sequencing efforts can play in development of novel markers of MPNST biology. 

### 6.5. ATRX Mutation and Evidence for Alternative Lengthening of Telomeres

In addition to the role of PRC2 in MPNST chromatin regulation, the chromatin regulator ATRX (Alpha Thalassemia/Mental Retardation Syndrome X) has been identified as mutated in a subset of MPNST [75]. Loss of ATRX function is involved in alternative lengthening of telomeres (ALT), a telomerase-independent means of telomere maintenance which prevents tumor cell senescence and promotes tumorigenesis. Subsequent studies on a larger subset of MPNST identified decreased nuclear expression of ATRX and demonstrated a correlation between aberrant ATRX expression and decreased overall survival in NF1-associated MPNST [84]. In a separate study a small subset (*n* = 3) of NF1-associated MPNST that were ALT-positive were analyzed by NGS and found to have *ATRX* mutations in two out of three cases [76]. While this study did not identify inferior overall survival (OS) for ALT-positive MPNST compared to those with normal telomere length, short telomeres were significantly correlated with improved OS. 

### 6.6. Beyond SUZ12: Less Common Variant Mutations in Other Chromatin Modifying Genes 

In addition to loss of *SUZ12* and *EED*, several studies have demonstrated additional alterations in PRC2 components or associated chromatin modifying genes. Sohier and colleagues detected a novel sequence change in the histone lysine demethylase *KDM2B* by WES (c3376C > T) in one out of eight human MPNST. This change is thought to potentially impact protein function; in an additional set of 14 tumors assayed by qPCR, KDM2B expression was reduced [44]. Whole genome and whole exome sequencing on an additional subset of NF1-associated MPNST identified mutations in additional chromatin associated genes including *CHD4*, *AEBP2*, *EPC1*, and *EZH2*, particularly in tumors with intact *SUZ12* [42].

### 6.7. Evidence for Alterations in the HIPPO Pathway in a Subset of MPNST and Schwann Cell Derived Tumors

Several studies have found evidence for alterations in the HIPPO–YAP pathway in MPNST. Analysis of aCGH from 51 MPNST samples [40] revealed an increase in the copy number of HIPPO effector gene loci, including *TAZ*, *CTGF* and *BIRC5* and a loss of HIPPO inhibitory gene loci, such as *LATS2* and *AMOTL2* [77]. In agreement with these findings, transcriptome sequencing of human MPNST samples from two additional patient cohorts revealed elevated YAP-activated gene expression in MPNST relative to normal nerves and NF1-associated neurofibromas [85,86]. Genomic alterations in the HIPPO pathway appear to occur in additional NF1 patient tissues including somatic mutations in seven of 33 cNF described in a recent study and as germline mutations (e.g., missense, frameshift and occasionally insertion) in seven of nine NF1 patients from the same dataset [87]. Together these studies validate the role of HIPPO pathway in neurofibroma biology and as a driver of MPNST tumorigenesis [77,87].

## 7. Beyond Genomics: The State of Understanding MPNST Transcriptomes, Proteomes, Epigenomes, and Metabolomes

In addition to the genomic alterations described above, these and other studies on human MPNST have revealed downstream effects on MPNST gene product expression and signaling. These investigations have confirmed or supplemented the genomic data by assaying downstream pathway effects in human MPNST. Several studies have broadly analyzed gene expression in human MPNST samples using microarray or RNAseq approaches [48,88]; these data can be examined in relation to known genetic changes to generate additional hypotheses for effects on downstream signaling pathways. Recent work compared gene expression in multiple functional pathways across pNF, ANF/ANNUBP, and MPNST and found that some ANNUBP share signaling pathway characteristics that more closely resemble pNF (e.g., ERK/MAPK) and others (e.g., AKT/mTOR) are more similar to MPNST [88]. Phospho-proteome arrays may be used to investigate kinase signaling in relation to various genomic alterations or therapeutic interventions in MPNST; to date this has primarily been used in MPNST cell lines or animal models (see article by Grit et al. in this Special Issue on Genomics and Models of Nerve Sheath Tumors) [89]. Methylation analysis on MPNST has revealed overall decreased histone and DNA methylation [90], and has also revealed how methylation changes in MPNST can affect expression of other tumor suppressor genes (e.g., *PTEN*) in MPNST [91]. Parallel methylation analysis and proteomic analysis on a set of nine MPNST samples characterized the relationship between PRC2 LOF on histone and DNA modification and consequent gene product expression. This work found that PRC2 loss was associated with increased pro-growth and immune evasion protein expression [92]. To date global metabolomics profiling has not been reported on human MPNST specimens; several recent efforts have examined metabolic shifts in animal models of MPNST in response to preclinical therapeutic interventions [93,94,95]. 

## 8. Translating Molecular Landscape of MPNST into Improved Therapies for Patients

One overarching goal of improved molecular characterization of MPNST is to translate genomic discoveries into improved treatments for this classically chemo-refractory tumor. As a result of improved understanding of MPNST genomic variants, several targeted therapies have been trialed in preclinical MPNST models. For example, the MET-specific tyrosine kinase inhibitor capmatinib has shown promise, particularly in combination with the MEK inhibitor trametinib, in an NF1-MET driven MPNST GEM model [70]. BRAF mutant MPNST may also respond to targeted therapy; one case report described a dramatic response to the RAF inhibitor vemurafenib in a patient with sporadic metastatic MPNST harboring the BRAF V600E mutation [96]. Efforts to target histone acetylation in a preclinical MPNST model with loss of SUZ12 shrank tumors when combined with MEK inhibition [41], while other DNA methyltransferase inhibitors appear to affect immune surveillance of MPNST [92]. It is likely that in the near future MPNST clinical trials will incorporate therapies inhibiting components of the epigenetic machinery. 

## 9. Conclusions

Significant research efforts over the past three decades have significantly advanced the state of knowledge of the genetic landscape of human MPNST. Particularly in NF1-associated MPNST, it is generally accepted that alterations in *NF1*, *CDKN2A*, *TP53*, and *SUZ12* are involved in tumor progression from benign to malignant tumors. However, less frequent alterations in genes with complementary function have been described in subsets of tumors, and additional tumor-driving mutations may be present in sporadic or recurrent/metastatic tumor samples. Future genomic studies should aim to incorporate as many well-annotated samples as feasible and clearly report on differences between NF1-associated and sporadic MPNST subtypes. Exciting future work will also incorporate additional technologies to improve our understanding of the downstream consequences of genomic alterations for MPNST biology and aid in development of improved treatments. 

## Figures and Tables

**Table 1:** Genomic sequencing studies for most common genetic alterations in human MPNST. All MPNST or neurofibromas in study reported under *n*. Reported sequencing results given as in reference (cases or percentages) for human MPNST specimens.

Ref.	Study Author Year	Description	*n* Total MPNST (*n* NF1 Associated) *n* (Other Specimen Types)	*NF1*	*CDKN2A*	*TP53*	*EED*	*SUZ12*	Notes
[38]	Mantripragada, 2008	Targeted seq, aCGH	35 (35) 16 pNF 8 cNF	71%	39%	17%	NR	NR	
[39]	Verdijk, 2010	Targeted seq	88 (26)	NR	ND	17/72	ND	ND	36% of *TP53* mutations detected were from NF1 patients
[40]	Yang, 2011	aCGH	51 (16)	~30%	65%	~30%	NR	NR	
[41]	DeRaedt, 2014	Targeted seq, aCGH	51 (51)	51/51	NR	NR	15/51	32/51	
[42]	Zhang, 2014	WGS (5), WES (3), Targeted seq (42)	50 (39) 11 (paired neurofibroma)	22/50	1/8	1/8	1/50	16/50	
[43]	Lee, 2014	WES (15), SNP, targeted (37)	52 (27) 7 neurofibromas	45/52	42/52	23/52	19/52	25/52	RNAseq analysis of MPNST with PRC2 loss vs. intact PRC2 demonstrates enrichment of genes associated with development and morphogenesis
[44]	Sohier, 2017	Exome seq, aCGH	8 (8) 1 pNF 7 cNF	8/8	5/8	1/8	2/8	7/8	No *TP53* point mutations identified
[45]	Brohl, 2017	WES + SNP	5 (4) + 7 TCGA cases (6)	11/12	7/12	6/12	4/12	5/12	5/12 MPNST contain somatic Ras-pathway activating mutation
[46]	Zehir, 2017	IMPACT NGS	11	2/11	6/11	NR	1/11	2/11	Data accessible through cBioPortal
[47]	Kaplan, 2018	Foundation Medicine NGS 2014–2016	186 (clinical data NR)	102 of 186	57% overall (71% *NF1*-altered, 80% *BRAF* altered, 34% non-*NF1*/non-*BRAF* altered)	32% of *NF1* 14% of non-*NF1*	8% of *NF1*-altered, 13% of *BRAF*-altered, 3% of non–NF1/non–*BRAF*-altered	20% of *NF1*-altered, 13% of *BRAF*-altered, 9% of non–*NF1*/non–*BRAF*-altered	Data reported as % of NF1/BRAF cohorts rather than absolute numbers
[48]	Pemov, 2019	NF1 deep sequencing (4); WES (3); CNV (28)	31 (4) 16 ANF	4/4; 10/28 (Loss, CNV)	4/4; 20/28 (Loss, CNV)	0/3 (WES); 10/28 (Loss, CNV)	1/3 (WES); 10/28 (Loss, CNV)	1/3 (WES); 9/28 (Loss, CNV)	RNAseq reported for ANF and 4 MPNST
[49]	Pollard, 2020	WES	1 (1) 7 pNF 13 cNF	1	0/1	0/1	0/1	1/1	RNAseq on cNF, pNF, and MPNST samples from 23 patients

aCGH = array comparative genomic hybridization; WGS = whole genome sequencing; WES = whole exome sequencing; SNP = single nucleotide polymorphism; NGS = next generation sequencing; CNV = copy number variation; NR = not reported; ND = not determined; cNF = cutaneous neurofibroma; pNF = plexiform neurofibroma; ANF = atypical neurofibroma.

**Table 2:** Less frequent genomic alterations identified in MPNST.

Gene	Description	*n*	NF1	Altered	Details	Study	Ref.
*BRAF*	Targeted seq	18	NR	0	18 MPNST out of 1,320 nervous system tumors	Schindler, 2011	[68]
	Targeted seq	47	25	1	1/1 N581S	Bottillo, 2009	[32]
	Targeted seq	24	NR	0	0/24 MPNST with BRAF exon 15 mutation	Je, 2012	[69]
	Foundation NGS	186	102	10	5 of 10 BRAF V600E; 9 of 10 pathogenic; 1 of 10 VUS 47% with alteration in >/=1 non-NF1/non-BRAF gene in the *RAS/RAF* pathway (*ERBB2*, *ERBB3*, *ERBB4*, *KRAS*, *MET*, *HRAS*, *MAP2K1*, *MAP2K2*, *NRAS*). 7% with alteration in RTK (e.g., *KIT/PDGFR/FGFR1*)—some likely pathogenic 70% with alteration in DNA repair genes (*ATM*, *BARD1*, *BRCA1/2*, *FANCx*, *PBRM1*, *CHEK2*, *MSH2*, *MSH3*, *MSH6*, *NBN*, *PBRM1*, *POLE*, *RAD51*, *RAD51C*)	Kaplan, 2018	[47]
*MET*	WES	1	1	1 (amplified)	Single patient longitudinal sampling (pre/post treatment, recurrence, mets) Copy number alterations in *HGF*, *EGFR*	Peacock, 2018	[70]
	Targeted seq, aCGH	35		25% (amplified)	*HGF*, *EGR*, *PDGFRA* amplifications in 25-29% samples	Mantripragada, 2008	[38]
*EGFR*	aCGH	51		37% (19/51)	At least one EGFR pathway gene was altered in 84% of samples, including *GRB2*, *HRAS*, *MAPK1*, *STAT1*, and others.	Du, 2013	[71]
	Targeted gene sequencing	37	29/37	28% gain	Direct sequencing of *EGFR* exons 18–24	Holtkamp, 2008	[72]
	Targeted gene sequencing and FISH	27	14 of 25 pts	14 of 23 (copy number gain)	Direct sequencing of *EGFR* exons 18–21	Perrone, 2009	[73]
*IGF1R*	aCGH	51	16	24% (amplified)	>/= 1 gene in IGFR1 pathway altered in 82% cases	Yang, 2011	[40]
*AURKA*	SNP array, qPCR	13	NR	8	1/8 neurofibromas also with *AURKA* locus copy number increase	Patel, 2012	[74]
*TYK2*		7	7	2	Tyrosine kinase 2, activates STAT signaling and promotes cancer cell survival	Hirbe, 2017	[75]
*ATRX*	NGS clinical genomic profiling	7	7	NR		Hirbe, 2017	[75]
	NGS	4	4	2	Of 3 ALT-positive MPNST, 2 had *ATRX* mutations. One ALT positive MPNST had *RECQL4* variant.	Rodriguez, 2019	[76]
	WGS, WES	8	5	1	Additional chromatin organization-related genes: *EZH2*, *CHD4*, and *AEBP2* mutations *n* = 1 tumor each; *RBBP7* mutation in *n* = 2 tumors.	Zhang, 2014	[42]
*KDM2B*	Exome seq, aCGH	8	8	1	Jumonji histone lysine demethylase; identified in single patient MPNST lacking SUZ12 or EED mutation	Sohier, 2017	[44]
*LATS2*	aCGH	51	16	NR (copy number loss in ~25%)	Copy number gains and losses in HIPPO effector loci (*TAZ*, *CTGF*, *BIRC5*) and HIPPO inhibitory loci *(LATS2*, *AMOTL2*) graphically illustrated. Same dataset as Yang et al. Clin Can Res 2011.	Wu, 2018	[77]
*HMMR/RHAMM*	aCGH	35	71%	46%	Deletions in hyaluronan binding protein may affect signaling through ERK or AURAK	Mantripragada, 2008	[38]

NGS = next generation sequencing; aCGH = array comparative genomic hybridization; WGS = whole genome sequencing; WES = whole exome sequencing; SNP = single nucleotide polymorphism; NR = not reported; ND = not determined.
